# Natural products: promising therapeutics for targeting regulatory immune cells in the tumor microenvironment

**DOI:** 10.3389/fphar.2024.1481850

**Published:** 2024-11-13

**Authors:** Peng Song, Fei Song, Tingting Shao, Pengjuan Wang, Rongkun Li, Zhe-Sheng Chen, Zhaofang Zhang, Guozhong Xue

**Affiliations:** ^1^ Gansu University of Chinese Medicine, Lanzhou, China; ^2^ Affiliated Hospital of Gansu University of Chinese Medicine, Lanzhou, China; ^3^ College of Pharmacy and Health Sciences, St. John’s University, Queens, NY, United States

**Keywords:** regulatory immune cells, natural products, polysaccharides, polyphenols, tumor therapy

## Abstract

Regulatory immune cells regulate immune responses through various mechanisms, affecting the occurrence, development, and therapeutic effects of tumors. In this article, we reviewed the important roles of regulatory immune cells, such as regulatory T cells (Tregs), regulatory B cells (Bregs), myeloid-derived suppressor cells (MDSCs), regulatory dendritic cells (DCregs), and tumor-associated macrophages (TAMs), in the tumor microenvironment (TME). The immunomodulatory effects of natural products, such as polysaccharides, polyphenols, glycosides, alkaloids, terpenoids, quinones, and other compounds, which affect the functions of regulatory immune cells through molecular signaling pathways, thereby enhancing the potential of the antitumor immune response, are discussed. These findings provide new ideas and possibilities for the application of natural products in tumor treatment, which can help enhance the effectiveness of tumor treatment and improve patient prognosis.

## Highlights

• The role of regulatory immune cells in the tumor microenvironment was comprehensively discussed.
• A comprehensive overview of the classification and sources of natural products was provided.
• The mechanisms by which natural products act on regulatory immune cells are discussed.

## 1 Introduction

**FIGURE 1:**
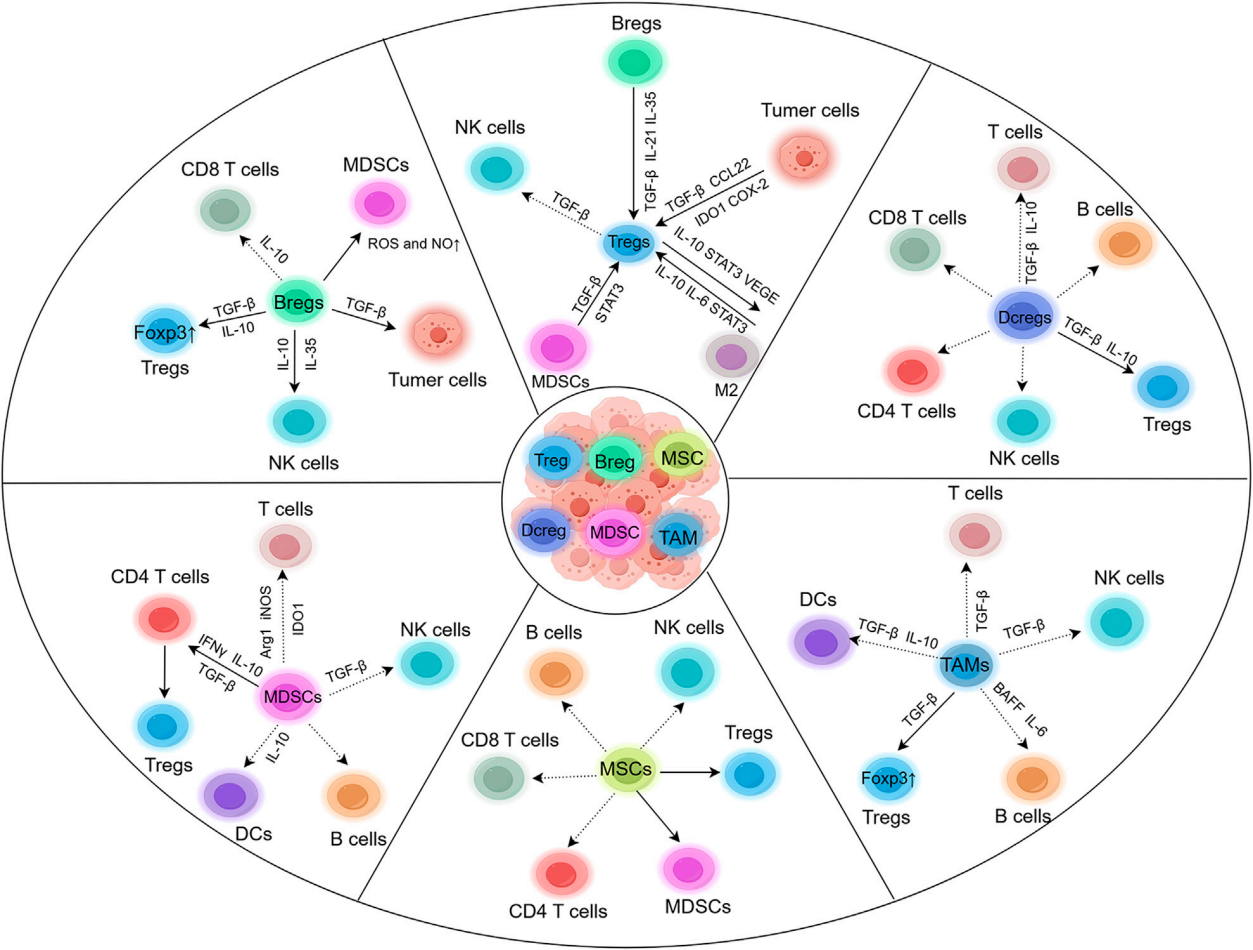
Types and functions of regulatory immune cells.

The tumor microenvironment (TME), which is composed of cancer cells, stromal cells, and immune cells, plays an important role in tumorigenesis (Ceglie et al., 2024). Regulatory immunological cells, including regulatory T cells (Tregs), regulatory B cells (Bregs), regulatory dendritic cells (DCregs), myeloid-derived suppressor cells (MDSCs), mesenchymal stem cells (MSCs), and tumor-associated macrophages (TAMs), abundantly infiltrate tumor tissues and suppress immunity, which is often associated with poor progression in cancer patients (Farbod et al., 2022). Studies have shown that regulatory immune cells can be selectively depleted or functionally attenuated to evoke an effective antitumor immune response. Furthermore, regulatory immune cells are characterized by high levels of immune checkpoints (María et al., 2023). Thus, immune checkpoint inhibitors that block related molecules enhance the immune response and attenuate the inhibitory activity of regulatory cells (María et al., 2023). An increasing number of studies indicate that regulatory immune cells are potential targets for immunotherapy in cancer patients. In this review, we discuss the relationships among different regulatory immune cells and the molecular basis of their behavior in tumor tissues to evoke effective antitumor immunity (Figure 1).

Cancer is one of the leading causes of death worldwide. While battling this disease in China and other East Asian countries, many traditional Chinese medicines (TCMs) are remarkably effective in treating patients with cancer (Xiang et al., 2019). TCM drugs are traditionally composed of many different components, such as polysaccharides, polyphenols, glycosides, alkaloids, terpenoids, and quinones, that can target various pathways to directly inhibit the growth of cancer and change the host immune system (Li et al., 2021). We summarize the latest progress in TCM-related natural components in cancer from theory to clinical practice, which could lead to the development of sophisticated TCM and promote the development of modern medicine.

## 2 The classification and function of regulatory immune cells

### 2.1 Regulatory T cells

Tregs, a subset of CD4^+^ T cells, are closely related to the pathogenesis of cancer and express the transcription factor FoxP3. FoxP3 plays a major role in maintaining immunological balance and is a vital factor in the immunosuppressive TME (Liu et al., 2024). Tregs have significant phenotypic and functional heterogeneity and are classified into three categories on the basis of the expression of *FoxP3* and its inhibitory effects. Stable Tregs, which express sustained *FoxP3*, *Nrp1*, and *Helios*, have immunosuppressive functions and constitute the main subpopulation of Tregs. Unstable Tregs, which have low expression of *FoxP3* and IFN-γ, support the antitumor immune response. Fragile Tregs are functionally less suppressive in the TME, which is defined as the retention of FoxP3 expression and the secretion of IFN-γ (Abigail and Vignali Dario, 2018). Tregs can inhibit antitumor immune responses and promote tumor growth and cross-talk with type 2 macrophages (M2), MDSCs, Bregs, and natural killer (NK) cells. Tregs inhibit NK cells by releasing TGF-β, IL-10, STAT3, and VEGF to mediate the differentiation of M2 macrophages. Tregs express PD-1 receptors on their surface and release STAT3 into the TME to inhibit the immune system, which leads to immune escape from cancer cells and a reduction in antitumor immune function (Najafi et al., 2019).

### 2.2 Regulatory B cells

Bregs are defined as all B cells that suppress immune responses and mediate the negative regulation of immune responses; these Bregs secrete cytokines to affect antitumor immune function and cancer cell growth directly and indirectly. Based on the cytokines that they secrete, Bregs are classified into four types. CD19^+^ CD25^hi^ Bregs can enhance the function of Tregs. CD24^+^ CD38^+^ Bregs express IL-10. CD19^+^ CD38^+^ CD1d^+^ IgM^+^ CD147^+^ Bregs express granzyme B (GrB), which is induced by IL-21. CD19^+^ CD24^+^ CD38^+^ Bregs exist in patients with invasive breast cancer (IBCa) (Tanja et al., 2019). Bregs exert immunomodulatory effects through cell-to-cell contact and cytokines. In the TME, Bregs inhibit effector T cells and target other tumor-infiltrating immune cells, such as Tregs, MDSCs, NK cells, and macrophages, to hinder antitumor immunity. Additionally, the cross-regulation of Bregs and tumor cells promotes cancer progression and helps tumors evade immune surveillance (Shang et al., 2020). Studies have shown that Bregs produce TGF-β to increase the expression of FoxP3 in Tregs and secrete IL-10 and IL-35 to inhibit the function of CD8^+^ T cells *in vitro*. In addition to directly inhibiting the function of effector T cells, Bregs indirectly convert CD4^+^ T cells into *FoxP3* ^+^ Tregs through TGF-β, which induces and promotes the production of an immune-suppressive microenvironment (Chen et al., 2019). At the same time, PD-L1 is constitutively expressed on B cells, called PD-L1 Bregs, which interact with PD-1 CD4^+^ T cells and PD-1 follicular helper T cells, leading to an inhibition of humoral immune responses. Of note, PD-L1 Bregs also decrease the production of pro-inflammatory cytokines and increase IL-10 to suppress the antitumor response (Catalán et al., 2021; Michaud et al., 2021).

### 2.3 Dendritic cells

DCregs, which are subsets of dendritic cells (DCs) with immunosuppressive activity in the TME, have strong immunosuppressive activity and are generally found in the microenvironment of advanced solid tumors (José et al., 2016). DCs include myeloid, lymphoid, conventional (cDCs), and plasma cell subsets (pDCs), which have specific immunostimulatory and immunosuppressive functions (Badillo et al., 2024). DCregs directly inhibit the activity of antitumor T cells by producing IL-10 and TGF-β and produce strong antigen-specific tolerance by inducing the differentiation of CD4^+^ Tregs. Additionally, IL-10 and TGF-β secreted by DCregs promote the transformation of CD4^+^ T cells into Tregs and enhance the immunosuppressive activity of natural Tregs. In conclusion, DCregs directly and indirectly induce regulatory responses by cross-talking with B cells, NK cells, and CD8^+^ T cells, thus inhibiting antitumor immunity (Verena et al., 2015).

### 2.4 Myeloid-derived suppressor cells

MDSCs are immature cells composed of myeloid progenitor cells, immature macrophages, immature granulocytes, and immature dendritic cells that affect tumor immunosuppression, angiogenesis, drug resistance, and metastasis (Chen H. et al., 2022). Based on their different surface markers, MDSCs can be divided into two subtypes: granulocytic MDSCs (PMN-MDSCs, also known as multinucleated MDSCs) and monocytic MDSCs (M-MDSCs). PMN-MDSCs constitute the main population of MDSCs (approximately 80%), which primarily inhibit antigen-specific CD8^+^ T cells by producing reactive oxygen species (ROS). M-MDSCs are rapidly transformed into tumor-associated macrophages (TAMs) in the hypoxic region of tumors to increase their immunosuppressive effect (Gabrilovich et al., 2012). PMN- and M-MDSCs not only produce Arg1, iNOS, and IDO1 to inhibit the functions of T cells but also secrete IFN-γ, IL-10, and TGF-β to convert CD4^+^ T cells into Tregs and impair the function of DCs by producing IL-10. MDSCs also exert immunosuppressive effects by impairing other immune cell functions, including those of B cells and NK cells (Wang et al., 2022).

### 2.5 Mesenchymal stem cells

MSCs are self-renewing pluripotent stem cells that were initially identified in the bone marrow; these cells regulate innate and adaptive immune responses and affect the antigen-presenting properties of DCs, B cells, and macrophages. Studies have shown that MSCs have the ability to regulate the phagocytic capacity of neutrophils and monocytes; change the polarization of macrophages and the cytotoxicity of NK cells; and regulate the proliferation, activation, and effector functions of CD4^+^ and CD8^+^ T cells (Harrell et al., 2021). MSCs also induce the generation of immunosuppressive Tregs and MDSCs in a paracrine manner and exert suppressive effects on CD8^+^ Cytotoxic T-lymphocytes (CTLs). Thus weakening antitumor immunity and enabling tumor growth and progression (Sarhan et al., 2020; Song et al., 2020).

### 2.6 Tumor-associated macrophages

TAMs, another type of tumor-infiltrating cell, originate from monocytes in the bone marrow and are among the key regulators of the immune response in the TME. After being exposed to the TME, macrophages undergo M1-like or M2-like polarization to promote or inhibit tumors (Zhang, et al., 2022). M1 macrophages promote a pro-inflammatory response, damage tissue integrity, dampen tumor progression, and are induced by T-helper type-1 cytokines, including IFN-γ, IL-1β, and lipopolysaccharides (LPSs). However, M2 macrophages, also called TAMs, are anti-inflammatory cells induced by IL-4 and IL-13 secreted by Th2 cells, which promote cancer cell proliferation, invasion, tumor metastasis, and angiogenesis and participate in immune suppression (Basak et al., 2023). TAMs inhibit the antitumor activity of lymphocytes through various mechanisms. On one hand, TAMs release TGF-β to directly inhibit the effector functions of T cells and NK cells. On the other hand, TAMs hinder the maturation of dendritic cells, promote the expansion of Tregs, and impair the functions of T cells by releasing the immunomodulatory enzymes arginase-1 (Arg^–1^), indole-2, and 3-dioxygenase (IDO) (MdNabiul et al., 2022).

## 3 Natural products that act on regulatory immune cells

### 3.1 Polysaccharides

Most TCM substances are derived from herbal plants, and polysaccharides are major components of these plants. In recent decades, polysaccharides isolated from different types of TCMs have received much attention in the treatment of cancer because of their multiple working pathways and minimal adverse reactions. The natural sources and comprehensive effects of polysaccharides on immune cells are listed in Table 1.

**TABLE 1:** Natural antitumor polysaccharides and their sources, experimental models, and mechanisms of targeting regulatory immune cells.

Name	Source	Tumor type	Clinical/experimental	Targeted regulatory immune cell mechanisms (drug concentration)	Reference
Polysaccharides from *Cistanche deserticola*	*Cistanche deserticola* Ma	Liver cancer	*In vitro*	Promoting the polarization of TAMs from the M1 type to the M2 type by activating the NF-κB pathway (500 μg/mL)	Cui Y. (2021)
Astragalus polysaccharides	*Astragalus membranaceus* (Fisch.) Bge	Non-small-cell lung cancer	*In vitro*	Decreasing the number of MDSCs by promoting the differentiation of PMN-MDSCs into granulocytes (20 mg/mL)	Liu (2018)
		Colon cancer	*In vitro*	Inhibiting the secretion of IL-10 and TGF-β through Tregs by downregulating the expression of CD4 and CD25 (0.5–50 µg)	Zuo (2019)
		Melanoma	*In vivo*	Inhibiting the secretion of TGF-β and IL-10 by reducing the number of Tregs in the spleens of melanoma mice (0.13 and 0.3 g/kg, respectively, of 0.2 mL once a day for 14 days, i.g.)	Sun et al. (2013)
		Melanoma	*In vivo*	Inhibiting the secretion of IL-10 and VEGF by reducing the proportion of MDSCs (150 mg/mL of 0.2 mL once a day for 14 days, i.g.)	Cai et al. (2012)
		Gastric cancer	Clinical	Inhibiting the progression of gastric cancer by reducing the expression of CD4^+^ CD25^+^ FoxP3^+^ Tregs (125 and 250 mg, respectively, once a day for 7 days, i.v.)	Lv et al. (2021)
		Liver cancer	*In vitro*	Decreasing the number of CD4^+^ CD25^+^ Tregs by inhibiting the expression of FoxP3 mRNA and the secretion of Th2 cytokines (100 μg/mL, 48 h)	Li et al. (2012)
		Lung cancer	*In vivo*	Decreasing the accumulation of MDSCs in the pre-metastatic niche of the lung by inhibiting the S1PR1/STAT3 pathway (50, 100, and 200 mg/kg, respectively, once a day for 28 days, i.g.)	Shen et al. (2023)
*Glycyrrhiza* polysaccharide	*Glycyrrhiza glabra*	Colon cancer	*In vitro*	Inhibiting the secretion of IL-10 and TGF-β through Tregs by downregulating the expression of CD4 and CD25 (0.5–50 µg)	Zuo (2019)
*Armillaria mellea* polysaccharides	*Armillaria mellea*	Colon cancer	*In vitro*	Inhibiting the polarization of M2 macrophages and promoting M1 polarization by activating the Akt/NF-κB, ERK/NF-κB, and JNK/NF-κB pathways (200 μg/mL)	Dan (2019)
Polysaccharide from *Dictyophora*	*Dictyophora*	Lung cancer	*In vivo*	Reducing the proportion of MDSCs in the spleens of lung cancer mice by upregulating P53 gene expression and downregulating Bcl-2 gene expression in MDSCs (25 mg/kg once a day for 25 days, i.p.)	Jiang and Wang (2019)
Polysaccharides from *Aconitum brachypodum* Diels	*Aconitum brachypodum* Diels	Liver cancer	*In vitro* and *in vivo*	Inhibiting lung metastasis of liver cancer mice by downregulating Tregs and promoting T-lymphocyte proliferation (250 and 500 mg/kg, respectively, once a day for 11 days, i.g.)	Peng et al. (2023)
Lentinan	*Lentinula edodes*	Bladder cancer	*In vivo*	Suppressing the progression of bladder cancer by inhibiting the secretion of IL-10 and TGF-β by MDSCs and Tregs (4 mg/kg twice a week for 35 days, i.p.)	Sun et al. (2020)
		Non-small-cell lung cancer	Clinical	Inhibiting the progression of non-small-cell lung cancer by suppressing the proliferation of Tregs (4 mg once a day for 12 weeks, i.m.)	Wang et al. (2018)
Fucoidan	Brown alga seaweed	Breast cancer	*In vivo*	Reducing the percentage of Tregs by inhibiting the PD-1/PD-L1 signaling pathway (200 and 400 mg/kg, respectively, six times a week for 4 months, i.g.)	Xue et al. (2017)
*Ganoderma lucidum* polysaccharide	*Ganoderma lucidum*	Liver cancer	*In vivo* and *in vitro*	Inhibiting Treg accumulation and function by increasing the expression of miR-125b (10, 50, 100, and 200 mg/kg of 0.5 mL once every 2 weeks for 4 weeks, i.p.)	Li et al. (2015)
		Lewis lung cancer	*In vivo*	Inducing the differentiation of MDSCs and inhibiting their accumulation by activating the CARD9-NF-κB-IDO pathway (25 and 100 mg/kg, respectively, once a day for 14 days, i.g.)	Wang et al. (2020)
*Grifola frondosa* polysaccharide	*Grifola frondosa*	Breast cancer	*In vivo*	Inhibiting the growth of breast cancer by eliminating MDSCs and enhancing T-cell responses (25, 50, and 100 mg/kg, respectively, of 100 µL every other day for 25 days, i.p.)	Li et al. (2024)
*Lachnum* polysaccharide	*Lachnum*	Sarcoma	*In vivo*	Reducing the aggregation of MDSCs and Tregs and promoting the transition of TAMs from the M2 to the M1 phenotype by activating the NF-κB pathway (200 mg/kg of 0.2 mL once a day for 20 days, i.g.)	Zong, Li, Ye, Zhang, Liu (2020)
Asparagus polysaccharide	*Asparagus officinalis* L	Colon cancer	*In vitro*	Inhibiting MDSC activity by enhancing TLR4 expression (0.5 mg/mL; IC_50_: 0.4919 mg/mL)	Zhang et al. (2018)
Modified citrus pectin	Citrus, lemon, orange, and grapefruit	Breast cancer	*In vivo* and *in vitro*	Inhibiting breast cancer development in mice by suppressing M2 polarization in the hypoxic microenvironment (0, 0.02%, 0.05%, and 0.1%, respectively, 72 h)	Wang et al. (2021)
Polysaccharide from *Ilex asprella*	*Ilex asprella*	Sarcoma	*In vivo* and *in vitro*	Suppressing M2 polarization by the NF-κB, STAT1, and STAT3 pathways (50 mg/kg, i.p.)	Qiu et al. (2018)
Polysaccharides of *Brassica rapa* L	*Brassica rapa* L	Lung cancer	*In vivo* and *in vitro*	Promoting M1 polarization by activating the STAT pathways (0.5, 1, and 2 g/kg, respectively, once a day for 10 days, i.g.)	Guo et al. (2022)

Astragalus polysaccharides are monomeric components extracted from Huangqi that have been widely studied for the treatment of non-small-cell lung cancer by inhibiting the S1PR1/STAT3 pathway and further promoting the differentiation of MDSCs in the peripheral blood (Liu, 2018; Shen et al., 2023). Additionally, Astragalus polysaccharides inhibit the recruitment of MDSCs and Tregs, which reduces the expression of IL-10, TGF-β, and VEGF (Cai et al., 2012). Some immunosuppressive cytokines expressed in tumors, such as IL-10 and TGF-β, are considered to be the main cause of failed antitumor immune responses (Sun et al., 2013; Wiguna and Walden, 2015; Zuo, 2019). Lentinan suppresses the progression of bladder cancer, accompanied by a significant reduction in IL-10 and TGF-β levels in MDSCs and Tregs (Sun et al., 2020). In addition, *Glycyrrhiza* polysaccharides also have the ability to inhibit the secretion of IL-10 and TGF-β in Tregs and downregulate the expression of CD4 and CD25 (Zuo, 2019). *Ganoderma lucidum* polysaccharides have recently been exploited as potential components in the treatment of cancer, which induce the differentiation of MDSCs and inhibit their accumulation by activating the CARD9-NF-κB-IDO pathway in Lewis lung cancer, thereby preventing cancer progression (Wang et al., 2020). *G. lucidum* polysaccharides also significantly suppress tumor growth in hepatoma-bearing mice, which is associated with an increase in the ratio of Teffs to Tregs through increased miR-125b expression (Li et al., 2015). The NF-κB and STAT pathways are central coordinators in innate and adaptive immune responses and play important roles in the control of malignant cells to resist apoptosis-based tumor surveillance (Fan et al., 2013).

**FIGURE 2:**
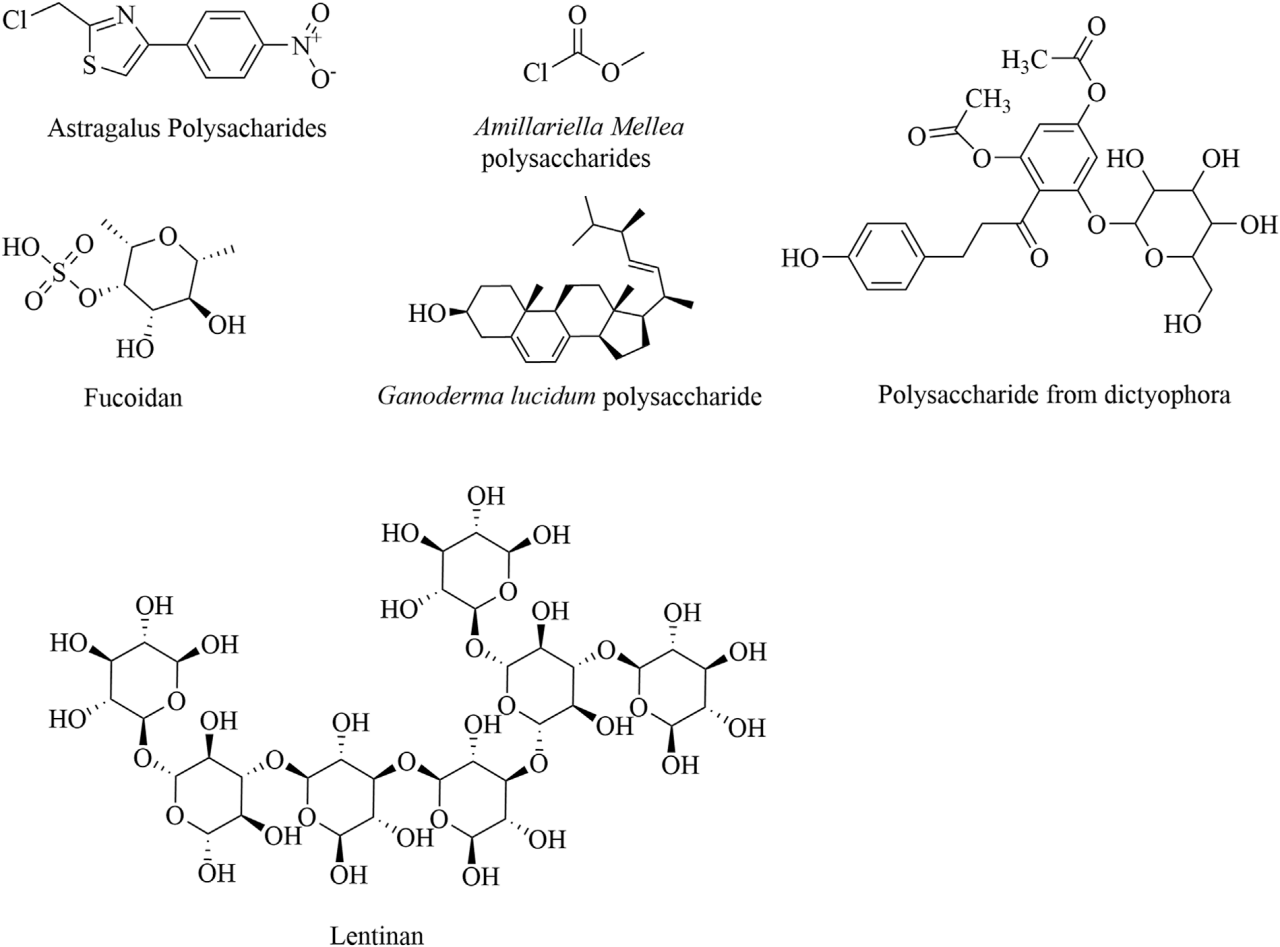
Structural formulas of natural polysaccharide products.

Most polysaccharides have been reported to inhibit tumor growth and reduce the aggregation of MDSCs, Tregs, and M2-TAMs through the NF-κB and STAT pathways. Examples include polysaccharides from *Armillaria mellea*, *Cistanche deserticola*, *Lachnum*, *Ilex asprella*, and *Brassica rapa* L (Cui Y. 2021; Dan, 2019; Guo et al., 2022; Qiu et al., 2018). *Dictyophora indusiata*, an edible mushroom, has demonstrated significant anticancer activity by enhancing immune function. It was found to significantly lower the proportion of MDSCs in the spleen of tumor-bearing mice by upregulating the expression of the pro-apoptotic P53 gene and downregulating the expression of the anti-apoptotic Bcl-2 gene (Jiang and Wang, 2019). Tregs play a crucial role in maintaining the balance between autoimmunity and immune suppression and are key in suppressing T-cell activation (Shan et al., 2022). Polysaccharides from *Aconitum brachiopod* Diels and fucoidan have been shown to reduce the percentage of CD3^+^ FoxP3^+^ Tregs while increasing the proportions of CD4^+^ and CD8^+^ T cells, providing experimental evidence of the immune enhancement effects induced by polysaccharides (Peng et al., 2023; Xue et al., 2017). The structural formulas of these polysaccharide products are given in Figure 2.

### 3.2 Polyphenols

**FIGURE 3:**
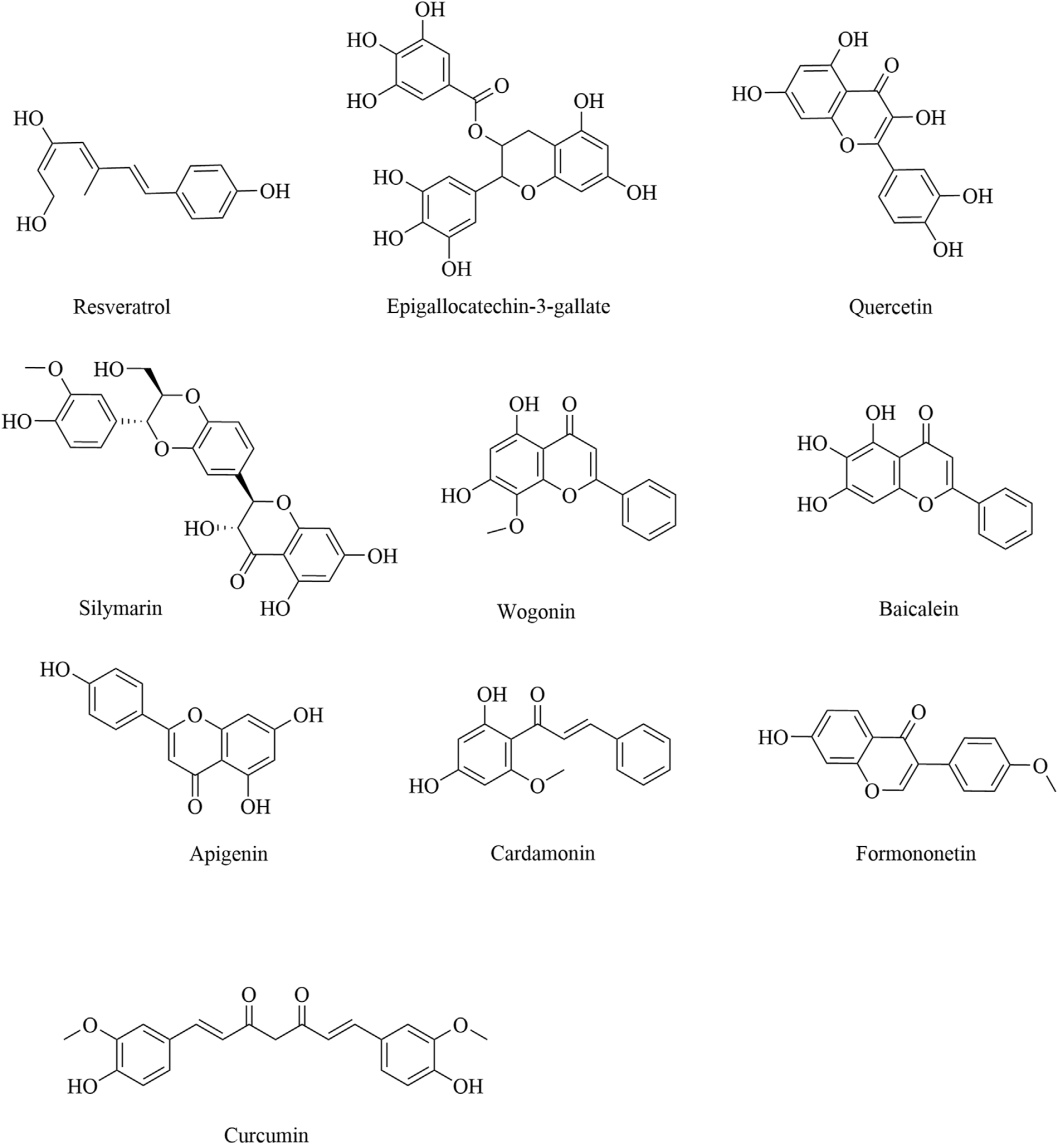
Structural formulas of natural polyphenol products.

Numerous studies have reported that natural polyphenols present in foods and beverages of plant origin have promising anticancer effects through the targeting of regulatory immune cells (Table 2). Based on their chemical structure, natural polyphenols can be divided into six classes: stilbenes, flavanols, flavonols, flavones, chalcones, and isoflavones. Resveratrol, a polyphenol found in grapes and blueberries, is widely recognized as an antitumor compound that modulates inflammation (Inoue and Nakata, 2015) and can inhibit tumor growth by increasing the percentages of CD8^+^ T cells, NK cells, and NK T cells and decreasing the percentages of Tregs, tBregs, and MDSCs (Catalina et al., 2013; Li et al., 2014; Tong et al., 2021; Yang et al., 2008; Zhou, et al., 2020). As discussed above, MDSCs are important factors in the TME. Resveratrol can promote not only the apoptosis of G-MDSCs but also the differentiation of M-MDSCs (Zhao, 2018; Zhao et al., 2019; Zhao et al., 2018). Recent research has shown that resveratrol exerts anticancer action by regulating the polarization state of TAMs and further inhibiting the migration and invasion of CAL27 cells by inhibiting the phosphorylation of the Syk protein (Li, 2022). A growing number of epidemiologic studies have shown an inverse relationship between cancer incidence and flavonoid intake. Epigallocatechin-3-gallate originates from green tea extract, which has the ability to downregulate canonical pathways in MDSCs, mainly through the Arg^–^1/iNOS/Nox2/NF-κB/STAT3 signaling pathway, and upregulate CD4^+^ and CD8^+^ T cells to ameliorate immunosuppression (Xu et al., 2020). Quercetin, silymarin, and *Radix Tetrastigma hemsleyani* flavones are flavonoid compounds that inhibit MDSCs (Hu, 2016; Hu et al., 2021; Ma et al., 2020; Wu et al., 2018). Notably, some flavanones, including wogonin, baicalein, and apigenin, can induce M1 macrophage polarization by activating the JAK2-STAT1 pathway and increasing the expression of SHIP-1 (Kazim et al., 2022; Xiao, 2021). Formononetin is a type of isoflavanone obtained from *Astragalus membranaceus*, whose therapeutic role in cancer involves blocking the PD-1/PD-L1 pathway to activate CD8^+^ T cells and reduce the release of immunosuppressive factors in Tregs (Li et al., 2023). Cardamonin is a dihydrochalcone isolated from *Elettaria cardamomum* with anticancer activity. Mechanistically, cardamonin suppresses the M2 polarization of TAMs and downregulates IL-10 and VEGF secreted by TAMs by inhibiting the phosphorylation of mTOR and STAT3 (Chen Z. et al., 2022). The anticancer potential of curcumin is observed in multiple cancer types, such as breast cancer and OSCC, and is associated with the suppression of the activity of Tregs and TAMs, as well as the downregulation of TGF-β and IL-10 by the MAO-A/STAT6 signaling pathway (Bhattacharyya et al., 2010; Jiang, 2023). The structural formulas of the polyphenolic products are shown in Figure 3.

**TABLE 2:** Natural antitumor polyphenols and their sources, experimental models, and mechanisms of targeting regulatory immune cells.

Category	Name	Source	Tumor type	Clinical/experimental	Targeted regulatory immune cell mechanisms	Reference
Stilbenes	Resveratrol	Grapes, berries, peanuts, and white hellebore	Oral squamous cell carcinoma	*In vitro*	Promoting macrophage polarization toward the M1 phenotype and inhibiting their polarization toward the M2 phenotype by activating the Syk pathway (20 μM, 24 h)	Li (2022)
			Lewis lung cancer	*In vivo*	Inhibiting tumor growth in Lewis lung cancer mice by promoting the apoptosis of G-MDSCs and the differentiation of M-MDSCs (50 mg/kg of 200 µL once a day for 3 weeks, i.g.)	Zhao et al. (2019)
			Lewis lung cancer	*In vitro* and *in vivo*	Inhibiting tumor growth in Lewis lung cancer mice by reducing the number of G-MDSCs (50 mg/kg of 200 µL once a day for 3 weeks, i.g.)	Zhao (2018)
			Osteosarcoma	*In vivo*	Inhibiting tumor growth in osteosarcoma mice by reducing the percentage of MDSCs and Tregs (50 and 100 mg/kg, respectively, once a day for 16 days, i.p.)	Tong et al. (2021)
			Liver cancer	*In vivo*	Inhibiting the generation of Tregs by downregulating STAT3 phosphorylation and reducing miR-21 expression (5 and 10 mg/kg, respectively, once a day for 15 days, i.g.)	Li et al. (2014)
			Breast cancer	*In vivo*	Inhibiting the metastasis of breast cancer by inducing the inactivation of tBregs (20 and 50 µg, respectively, every other day for 19 days, i.p.)	Catalina et al. (2013)
			T-cell lymphoma	*In vivo* and *in vivo*	Inhibiting tumor growth in T-cell lymphoma mice by reducing the number of Tregs (4 mg/kg once a day for 20 days, i.p.)	Yang et al. (2008)
			Liver cancer	*In vivo* and *in vitro*	Exerting antitumor effects by downregulating CD8^+^ CD122^+^ Tregs in murine hepatocellular carcinoma mice (50 mg/kg once a day for 3 weeks, i.g.)	Zhou et al. (2020)
			Lewis lung carcinoma	*In vivo*	Inhibiting tumor growth in Lewis lung carcinoma-bearing mice by decreasing G-MDSC accumulation and impairing its suppressive ability (5 mg/mL of 200 µL once a day for 3 weeks, i.g.)	Zhao et al. (2018)
			Hepatocellular carcinoma	*In vivo*	Inhibiting tumor growth in hepatocellular carcinoma mice by downregulating Tregs (50 mg/kg once a day for 3 weeks, i.g.)	Zhou et al. (2020)
Flavanols	Epigallocatechin-3-gallate	*Camellia sinensis*	Breast cancer	*In vivo* and *in vitro*	Promoting MDSC apoptosis by inhibiting the Arg-1/iNOS/Nox 2/NF-κB/STAT3 pathway (250, 500, and 1,000 μg/mL for 1 month)	Xu et al. (2020)
Flavanonols	Quercetin	Fruits, leaves, and seeds of plants	Colorectal cancer	*In vitro*	Blocking M2 macrophage polarization by inhibiting the FAM198B pathway and targeting the SMAD2 pathway	Zheng (2023)
			Prostatic cancer	*In vitro*	Enhancing the survival rate of G-MDSCs by activating the ESR/STAT3 pathway (20, 40, and 80 µM, respectively, for 24 h, 48 h, or 72 h)	Ma et al. (2020)
	Silymarin	*Silybum marianum* (L.) Gaertn	Lung cancer	*In vitro* and *in vivo*	Inhibiting tumor growth in Lewis lung cancer mice by reducing the proportion of MDSCs in the tissue (25 and 50 mg/kg, respectively, once a day for 15 days, i.g.)	Wu et al. (2018)
Total flavone	*Radix Tetrastigma hemsleyani* flavones	*Tetrastigma hemsleyanum* Diels Gilg	Lung cancer	*In vivo*	Inhibiting tumor growth in Lewis lung cancer mice by downregulating the expression of MDSCs, COX-2, and PEG2 (25, 50, and 100 mg/mL, respectively, of 0.2 mL once a day for 14 days, i.g.)	Hu et al. (2021)
			Lung cancer	*In vitro* and *in vivo*	Reducing the expression of CD152 in Tregs by decreasing the expression levels of PGE2 and COX-2 (50, 100, and 300 mg/mL, respectively, of 0.3 mL once a day for 14 days, i.g.)	Zhang (2014)
			Lung cancer	*In vitro*	Downregulating the expression of MDSCs by inhibiting the secretion of COX-2, PGE2, Arg-1, and iNOS in the peripheral blood (25, 50, and 100 mg/mL of 0.2 mL once a day for 14 days, i.g.)	Hu (2016)
Flavanones	Wogonin	*Scutellaria baicalensis* Georgi	Lung cancer	*In vitro* and *in vivo*	Inducing macrophage polarization to the M1 type by activating the JAK2-STAT1 pathway (80 mg/kg once a day for 17 days, i.g.)	Xiao (2021)
	Baicalein	*Scutellaria baicalensis* Georgi	Lung cancer	*In vitro* and *in vivo*	Inducing macrophage polarization to the M1 type by activating the JAK2-STAT1 pathway (80 mg/kg once a day for 17 days, i.g.)	Xiao (2021)
	Apigenin	*Apium graveolens* L	Pancreatic cancer	*In vivo*	Increasing the proportion of M1 macrophages in TAMs by enhancing the expression of SHIP-1 (25 mg/kg of 100 µL once a day for 16–17 days, i.p.)	Kazim et al. (2022)
Dihydrochalcones	Cardamonin	*Elettaria cardamomum*	Breast cancer	*In vitro*	Inhibiting M2 polarization by suppressing the expression of mTOR and the phosphorylation of STAT3 (20 ng/mL)	Chen (2022)
Isoflavanones	Formononetin	*Astragalus membranaceus* (Fisch.) Bge	Liver cancer	*In vitro* and *in vivo*	Inhibiting the secretion of IL-10 and TGF-β in Tregs by blocking the PD-1/PD-L1 pathway (10 and 50 mg/kg, respectively, three times a week for 28 days, i.p.)	Li et al. (2023)
Other phenolic compounds	Curcumin	*Curcuma wenyujin* Y. H. Chen et C. Ling, *Curcuma longa* L., and *Curcuma kwangsiensis* S. G. Lee et C. F. Liang	Oral squamous cell carcinoma	*In vitro*	Inhibiting the invasion and metastasis of oral squamous cell carcinoma by upregulating the expression of IL-12 and downregulating the expression of IL-10, iNOS, TNF-α, and Arg-1 in TAMs (5, 10, and 20 µM)	Liu et al. (2020)
			Oral squamous cell carcinoma	*In vitro*	Promoting the polarization of TAMs from the pro-tumor M2 phenotype to the antitumor M1 phenotype by inhibiting the MAO-A/STAT6 signaling pathway (20 µM)	Jiang (2023)
			Breast cancer (EAC)	*In vivo* and *in vitro*	Inhibiting the suppressive activity of Tregs by downregulating the expression of TGF-β and IL-10 (50 mg/kg every alternate day for 14 days, i.p.)	Bhattacharyya et al. (2010)

### 3.3 Saponins

**FIGURE 4:**
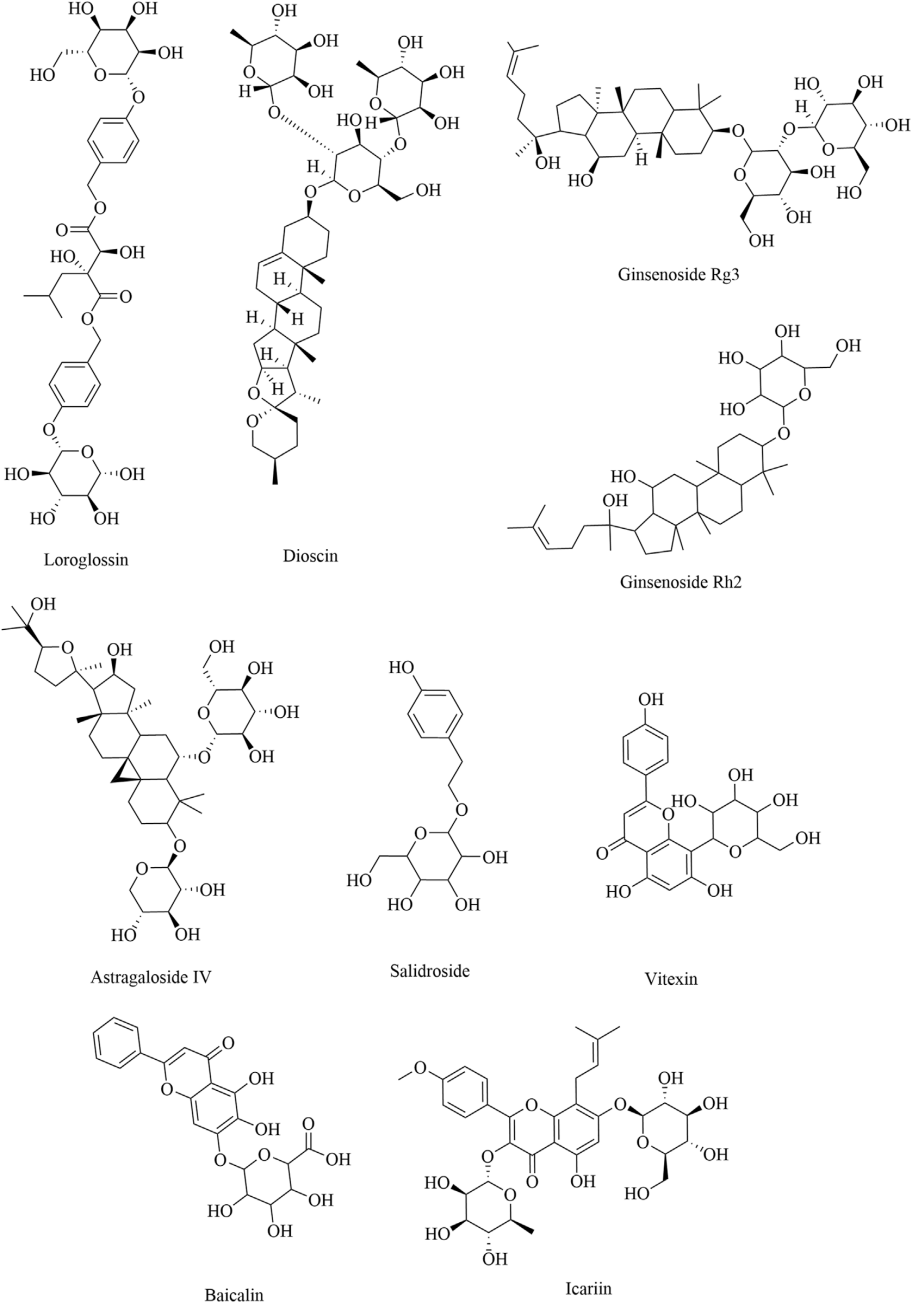
Structural formulas of natural saponin products.

Saponins consist of sapogenin and sugar molecules. Sapogenins are triterpenes or spirostanes, and the make-up sugars are commonly glucose, arabinose, rhamnose, galactose, and xylose. Many traditional Chinese herbs, such as *Paeonia lactiflora*, *Panax ginseng*, *A. membranaceus*, *Epimedium Tourn*, and *Rhodiola crenulata*, contain glycosides as their primary active ingredients. Owing to the structures of sapogenins, saponins exhibit biological activities such as antitumor, immune-regulatory, and anti-inflammatory effects. The natural sources and comprehensive influence of saponins on immune cells are listed in Table 3. B-cell activating factor (BAFF), a cytokine belonging to the TNF family, plays a crucial role in regulating the survival and differentiation of B cells. The total glucosides of *Paeonia* can reduce the proportion of Bregs by inhibiting the BAFF-BAFF-R signaling pathway (Yuan, 2015). Dioscin is a glycoside saponin isolated from *Dioscorea zingiberensis* and *Dioscorea nipponica* that can inhibit the polarization of M2 macrophages in TAMs by activating the Cx43/STAT1/IFN-γ pathway and inhibiting the IL-4/JAK2/STAT3 pathway (Cui et al., 2020; Sun, 2022). Ginsenoside Rg3 and ginsenoside Rh2, which originate from *P. ginseng* C. A. Mey, can inhibit tumor growth and metastasis in Lewis lung cancer mice by polarizing TAMs from M2 to M1 (He et al., 2012; Jiang, 2018; Li et al., 2018). Moreover, an increasing number of studies have reported that ginsenoside Rg3 exerts anticancer effects by inhibiting MDSCs, which is related to the suppression of the STAT3 and NOTCH signaling pathways (Song et al., 2020). Vitexin, a flavonoid-like compound derived from *Vitex negundo* L. var., promotes the polarization of M1 macrophages and inhibits M2 polarization by increasing the expression of STAT1/p-STAT1, thereby inhibiting tumor growth in colon and breast cancer mice (X. Yuan, 2019; Yuan et al., 2022). Tregs are crucial for inducing acquired tolerance to tumors and suppressing various immune responses. Astragaloside IV and salidroside can reduce tumor growth in Lewis lung cancer mice by weakening the immune function of Tregs (Li B. et al., 2022; Zhang et al., 2014). The structural formulas of saponin products are shown in Figure 4.

**TABLE 3:** Natural antitumor saponins and their sources, experimental models, and mechanisms of targeting regulatory immune cells.

Category	Name	Source	Tumor type	Clinical/experimental	Targeted regulatory immune cell mechanisms	Reference
Glycosides	Total glucosides of *Paeonia*	*Paeonia lactiflora* Pall	Liver cancer	*In vitro* and *in vivo*	Downregulating the proportion of Bregs by inhibiting the BAFF-BAFF-R pathway (30, 60, and 120 mg/kg, respectively, once a day for 4 weeks, i.g.)	Yuan (2015)
	Loroglossin	*Spiranthes sinensis* (Pers.)	Lung cancer	*In vitro*	Inducing MDSC apoptosis by activating the Bcl-2 pathway (0.3–1.2 mg/mL)	Qin and Pang (2019)
	Dioscin	*Dioscorea zingiberensis* and *Dioscorea nipponica*	Melanoma	*In vitro*	Promoting the polarization of TAMs from the M2 phenotype to the M1 phenotype by activating the Cx43/STAT1/IFN-γ pathway and inhibiting the IL 4/JAK2/STAST3 pathway (1 µM and 2 µM, respectively)	Sun (2022)
			Lung cancer	*In vivo*	Inhibiting the polarization of M2 in TAMs by downregulating JNK and STAT3 pathways (60 mg/kg once a day for 21 days, i.g.)	Cui et al. (2020)
			Colitis-associated colorectal cancer	*In vivo* and *in vitro*	Inhibiting tumorigenesis of colitis-associated colorectal cancer by promoting M1 and inhibiting M2 macrophage polarization and promoting the differentiation of MDSCs into M1-like and inhibiting its differentiation into M2-like macrophages *in vitro* (1.5, 5, and 10 mg/kg, respectively, once every 2 days for 11 weeks, i.g.)	Xun et al. (2023)
Steroidal saponins	Ginsenoside Rg3	*Panax ginseng* C. A. Mey	Lung cancer	*In vitro* and *in vivo*	Inhibiting tumor growth and metastasis in Lewis lung cancer mice by suppressing the infiltration of TAMs into the tumor stroma (10 mg/kg of 0.2 mL once a day for 24 days, i.p.)	He et al. (2012)
			Gastric cancer	*In vitro* and *in vivo*	Inhibition tumor growth in gastric cancer mice by inducing macrophage polarization to the M1 phenotype (3 mg/kg once every 2 days)	Jiang (2018)
			Breast cancer	*In vivo* and *in vitro*	Exerting anticancer effects by inhibiting MDSCs, which is related to the suppression of STAT3 and NOTCH signaling pathways (2.5, 5, and 10 mg/kg, respectively, five times a week for 3 weeks, i.g.)	Song et al. (2020)
	Ginsenoside Rh2	*Panax ginseng* C. A. Mey	Lung cancer	*In vivo*	Inhibiting lung cancer cell migration by polarizing TAMs from the M2 phenotype to the M1 phenotype (40 mg/kg once a day for 21 days, i.p.)	Li et al. (2018)
Tetracyclic triterpenoid saponins	Astragaloside IV	*Astragalus membranaceus* (Fisch.) Bge	Lung cancer	*In vivo* and *in vitro*	Inhibiting tumor growth in Lewis lung cancer mice by downregulating Tregs (40 mg/kg once a day until death, i.g.)	Zhang et al. (2014)
Phenolic glycosides	Salidroside	*Rhodiola crenulata* (Hook. f. et Thoms.) H. Ohba	Lung cancer	*In vitro* and *in vivo*	Inhibiting tumor growth in Lewis lung cancer mice by downregulating the function of CD4^+^ CD25^+^ FoxP3^+^ Tregs (6 mg/kg of 200 µL once a day for 18 days, i.p.)	Li P. et al. (2022)
Flavonoids	Vitexin	*Crataegus pinnatifida* Bge. and *Vitex negundo* L. var. *cannabifolia* (Sieb. et Zucc.) Hand. -Mazz	Colon cancer	*In vivo*	Inhibiting tumor growth in colorectal cancer mice by promoting M1 polarization of macrophages in the TME (20 and 40 mg/kg, respectively, once a day for 14 days, i.g.)	Yuan et al. (2022)
			Colon cancer and *in situ* breast cancer	*In vitro* and *in vivo*	Promoting macrophage M1 polarization and inhibiting M2 polarization by enhancing the expression of STAT1/p-STAT1 (20 and 40 mg/kg, respectively, once a day for 13 days, i.g.)	Yuan (2019)
	Baicalin	*Scutellaria baicalensis* Georgi	Lung cancer	*In vitro* and *in vivo*	Inducing macrophage polarization to the M1 type by activating the JAK2-STAT1 pathway (80 mg/kg once a day for 17 days, i.g.)	Xiao (2021)
	Icariin	*Epimedium* Tourn. ex L.	Colorectal cancer	*In vitro*	Inhibiting tumor metastasis in colorectal cancer mice by promoting apoptosis and differentiation of MDSCs (30 and 60 µM, respectively, 72 h)	Ruan et al. (2023)

### 3.4 Alkaloids

**FIGURE 5:**
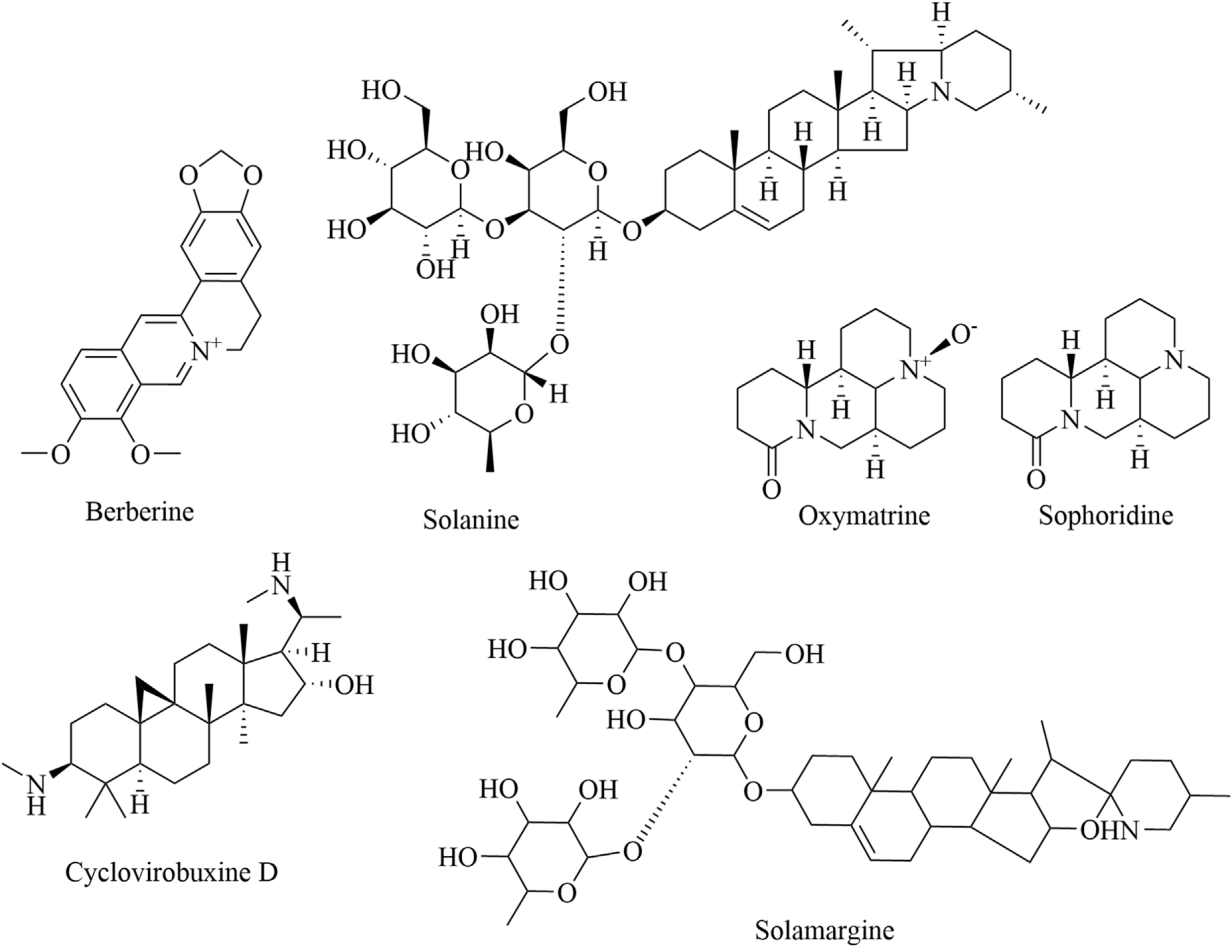
Structural formulas of natural alkaloid products.

Numerous studies have reported that natural alkaloids, whose heterocyclic moieties often exhibit improved solubility and can facilitate salt formation, can be used for the treatment and prevention of cancer (Rampogu et al., 2022). Therefore, heterocyclic moieties are important for oral absorption and bioavailability (Xiong et al., 2015). On the basis of their chemical structure, alkaloids can be categorized into three groups: isoquinolines, piperidines, and steroid alkaloids (Table 4). Berberine is an eminent isoquinoline quaternary alkaloid of TCM. Recently, berberine has gained much attention for its pharmacological action in treating cancer (Ortiz et al., 2014), which not only reduces the formation of TAMs but also downregulates IL-10 and TGF-β (J. Li et al., 2011). Among plant derivatives with biological properties, sanguinarine has a broad range of therapeutic uses against lung cancer. Specifically, sanguinarine inhibits angiogenesis in lung cancer by preventing M2 macrophage polarization via the Wnt/β-catenin pathway, while it also induces the differentiation of MDSCs into macrophages and DCs through the NF-κB pathway (Li P. et al., 2022; Cui Y. J. 2021). Solanine, oxymatrine, and sophoridine, important and active components of piperidines, have been found to have anticancer effects on multiple cancer types through the inhibition of immunosuppression. Solanine, extracted from *Solanum tuberosum* L., may activate the antitumor immune response by downregulating Tregs via the TGF-β/SMAD pathway (Gao et al., 2020). Oxymatrine can also mediate the differentiation of T cells into Tregs (Liu et al., 2019). Sophoridine induces TAM polarization into the M1 type through the TLR4/IRF3 axis and exerts stronger pro-inflammatory effects through the upregulation of the expression of INOS, IFN-β, and IL-12α (Zhuang et al., 2020). Cyclovirobuxine D, a natural alkaloid derived from *Buxus sinica* var. *parvifolia*, regulates the TME in the macrophage phenotype by regulating the immune checkpoint Siglec-10 (Gu, 2022). Solamargine also alleviates the expression of IL-4, IL-6, IL-10, and IL-13 and promotes the expression of IL-12 and TNF-α by inhibiting the polarization of TAMs (Liang, 2018). The related structural formulas of the natural alkaloid products are shown in Figure 5.

**TABLE 4:** Natural antitumor alkaloids and their sources, experimental models, and mechanisms of targeting regulatory immune cells.

Category	Name	Source	Tumor type	Clinical/experimental	Targeted regulatory immune cell mechanisms	Reference
Isoquinolines	Berberine	*Captis* *chinensis* Franch	Colorectal cancer	*In vivo*	Inhibiting tumor growth in colorectal cancer mice by suppressing the formation of TAMs (100 mM of 0.2 mL once a day for 14 days, i.p.)	Li et al. (2011)
			Lung cancer	*In vitro*	Suppressing the proliferation, invasion, and migration of A549 cells by inhibiting the transformation of TAMs into M2 macrophages (20 µM)	Qiu et al. (2019)
	Sanguinarine	*Spatholobus suberectus* Dunn	Lung cancer	*In vitro* and *in vivo*	Inhibiting the M2 polarization of macrophages by suppressing the Wnt/β-catenin pathway (5 and 10 mg/mL, respectively, of 0.1 mL once a day for 21 days, i.p.)	Cui Y. J. (2021)
			Lewis lung cancer	*In vivo*	Downregulating the proportion of MDSCs by activating the NF-κB/p65 pathway (5 mg/kg of 200 µL every other day for 20 days, i.p.)	Li B. et al. (2022)
Piperidines	Solanine	*Solanum tuberosum* L.	Liver cancer	*In vivo*	Downregulating the proportion of Tregs by inhibiting the TGF β/Smad signaling pathway (37.5 mg/kg of 0.2 mL once a day for 16 days, i.p.)	Gao et al. (2020)
	Oxymatrine	*Sophora flavescens* Ait	Lung cancer	*In vitro*	Enhancing Treg differentiation by promoting the maturation of DCs (1 mg/mL, 48 h)	Liu et al. (2019)
	Sophoridine	*Sophora alopecuroides* L.	Gastric cancer	*In vitro*	Promoting macrophage M1 polarization and inhibiting M2 polarization by activating the TLR4/IRF3 pathway (0.5, 1, and 2 mg/mL, respectively, 12 h)	Zhuang et al. (2020)
Other alkaloids	Cyclovirobuxine D	*Buxus sinica* var. *parvifolia* M. Cheng	Colorectal cancer	*In vitro*	Inhibiting M2 phenotype polarization of macrophages by suppressing the activity of Siglec-10 (10 µM)	Gu (2022)
	Solamargine	*Solanum nigrum* L.	Nasopharyngeal carcinoma	*In vivo*	Promoting the polarization of TAMs from M2 to M1 by inhibiting the expression of IL-4, IL-6, IL-10, and IL-13 cytokines and promoting the expression of IL-12 and TNF-α cytokines (4 and 8 mg/kg, respectively, of 0.2 mL once every other day for 14 days, s.c.)	Liang (2018)

### 3.5 Terpenoids

**FIGURE 6:**
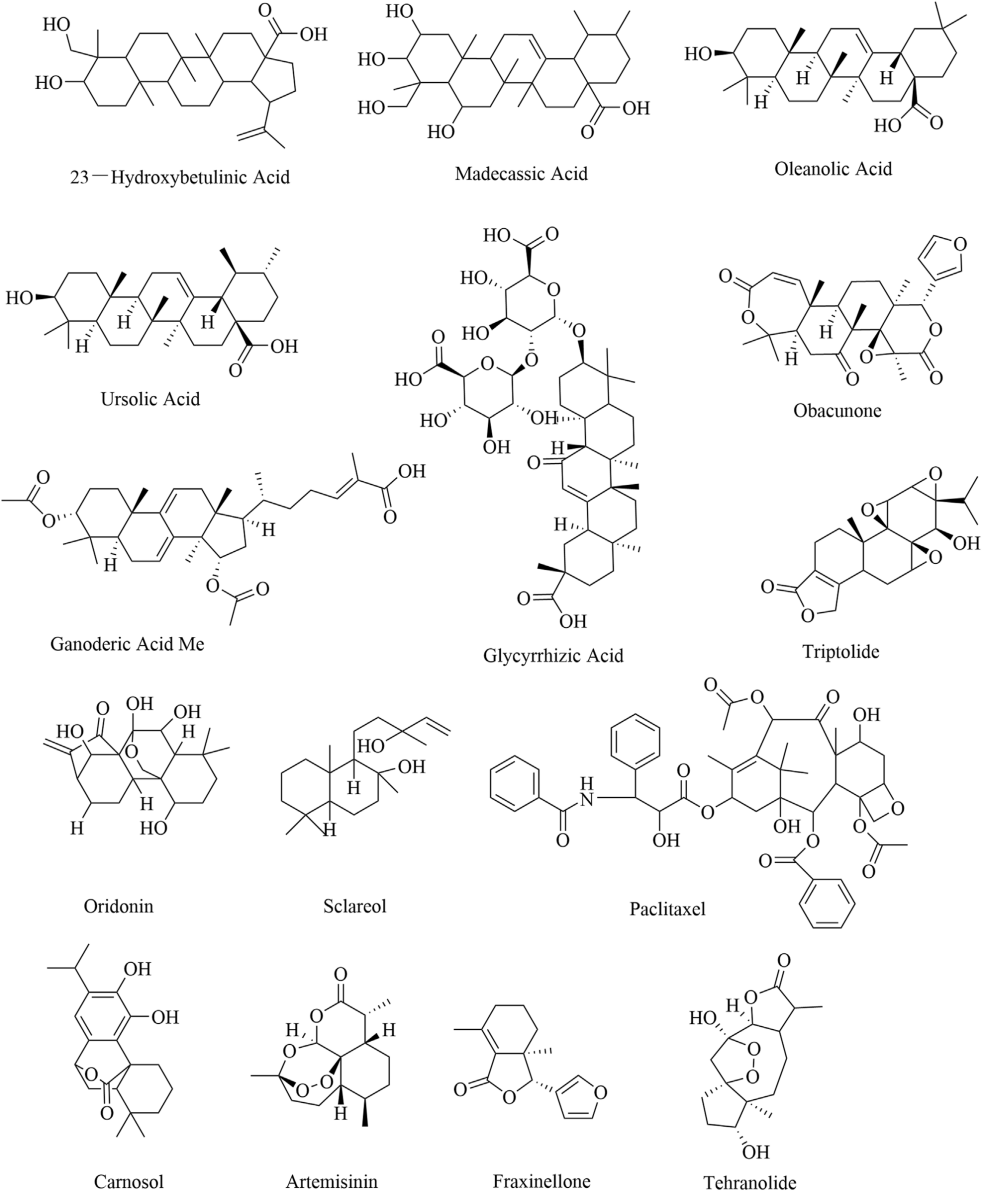
Structural formulas of natural terpenoid products.

Terpenoids are natural products derived from mevalonic acid and composed of multiple isoprene (C5) units with a general formula of (C_5_H_8_)n. Terpenoids are divided into three groups, namely, triterpenoids, diterpenoids, and sesquiterpenoids, based on their different molecular structures. The natural sources and comprehensive influences of terpenoids on immune cells are listed in Table 5. A triterpenoid compound, 23-hydroxybetulinic acid, isolated from *Pulsatilla chinensis* (Bge.) Regel, inhibits tumor growth in colon cancer mice by reducing the proportion of MDSCs and decreasing the expression of the immunosuppressive factor Arg1 in MDSCs (He et al., 2024). In a mouse model of colorectal cancer, 23-hydroxybetulinic acid can also promote the polarization of TAMs from the M1 phenotype to the M2 phenotype by activating the JAK/STAT3/NF-κB/STAT1 pathway (Liu, 2023). Madecassic acid alleviates colitis-associated colorectal cancer by blocking the recruitment of MDSCs via the inhibition of the expression of IL-17 in γδT17 cells (Yun et al., 2022). Moreover, oleanolic acid and ursolic acid are triterpenoid compounds that decrease the proportion of Tregs by inhibiting STAT5 phosphorylation and IL-10 secretion and promoting the expression of miR-98–5 (Xu et al., 2021; Zhang et al., 2020). Obacunone can also suppress tumor growth by promoting M1 macrophage polarization (Zou, et al., 2023). Notably, triptolide is the key diterpenoid extracted from *Tripterygium wilfordii* Hook. f., which reduces the proportion of Tregs and inhibits the secretion of IL-10 and TGF-β (Liu, 2011; Liu et al., 2013). Triptolide can also promote the apoptosis of MDSCs and the polarization of TAMs from M2 to M1 by inhibiting the PI3K/AKT/NF-κB pathway (Hu, 2020). Oridonin, paclitaxel, and carnosol inhibit the generation of Tregs by decreasing the expression of TGF-β and increasing the expression of CD95 and IFN-γ (Guo et al., 2020; Maryam, et al., 2015; Zhang et al., 2008). By promoting the differentiation of MDSCs into DCs, paclitaxel suppresses tumor growth in melanoma mice (Tillmann et al., 2012). The compounds of the sesquiterpenoids artemisinin and tehranolide, which are extracted from *Artemisia annua* L., inhibit tumor growth by reducing the number of Tregs (Cao et al., 2019; Noori, et al., 2009; Noori et al., 2010). The structural formulas of the terpenoid products are shown in Figure 6.

**TABLE 5:** Natural antitumor terpenoids and their sources, experimental models, and mechanisms of targeting regulatory immune cells.

Category	Name	Source	Tumor type	Clinical/experimental	Targeted regulatory immune cell mechanisms	Reference
Triterpenoids	23-Hydroxybetulinic acid	*Pulsatilla chinensis* (Bge.) Regel	Colon cancer	*In vitro* and *in vivo*	Inhibiting MDSC immunosuppressive function by the inhibition of MDSC differentiation, thereby restoring the antitumor activity of CD8^+^ T cells (7.5, 15, and 30 mg/mL, respectively, once a day for 18 days, i.v.)	He et al. (2024)
			Colorectal cancer	*In vitro* and *in vivo*	Promoting the polarization of TAMs from the M1 type to the M2 type by activating the JAK/STAT3/NF-κB/STAT1 pathway (15 and 30 mg/kg, respectively, once a day for 20 days, i.p.; 80 mg/kg once a day for 20 days, i.g.)	Liu (2023)
	Madecassic acid	*Centella asiatica* (L.) Urb	Colorectal cancer	*In vivo*	Increasing the population of antitumor immune cells in the tumor microenvironment by blocking the recruitment of MDSCs via the inhibition of the activation of γδT17 cells (6.25, 12.5, and 25 mg/kg, respectively, i.g.)	Yun et al. (2022)
	Oleanolic acid	*Pseudocydonia sinensis* (Dum.Cours.) C.K.Schneid	Gastric cancer	*In vivo*	Promoting the balance of Treg/Th17 cells by promoting the expression of miR-98–5 (NA)	Xu et al. (2021)
	Ursolic acid	Basil, apples, prunes, and cranberries	Breast cancer	*In vivo*	Modulating CD4^+^ CD25^+^ FoxP3^+^ T cells in 4T1 tumor-bearing mice by inhibiting STAT5 phosphorylation and IL-10 secretion (10 mg/kg once every other day for five times, i.v.)	Zhang et al. (2020)
	Glycyrrhizic acid	*Glycyrrhiza glabra*	Melanoma	*In vitro*	Inhibiting B16F10 cell proliferation by STAT3-mediated Treg and MDSC downregulation (NA)	Kumar et al. (2020)
	Ganoderic acid Me	*Ganoderma lucidum*	Lung cancer	*In vitro*	Enhancing Treg-mediated immunosuppression by directly inducing T-cell apoptosis and restraining CD8^+^ T-cell activation (NA)	Que et al. (2014)
	Obacunone	*Dictamnus dasycarpus* Turcz	Oral squamous cell carcinoma	*In vivo*	Suppressing tumor by promoting M1 macrophage polarization (50 and 100 mg/kg, respectively)	Zou et al. (2023)
Diterpenoids	Triptolide	*Tripterygium wilfordii* Hook. f	Liver cancer	*In vitro* and *in vivo*	Reducing the proportion of Tregs and inhibiting the secretion of anti-inflammatory factors such as IL-10 and TGF-β (0.157 and 0.314 mg/kg, respectively, once a day for 14 days, i.g.)	Liu (2011)
			Lung cancer	*In vitro* and *in vivo*	Inhibiting lung cancer by activating endoplasmic reticulum stress in MDSCs and promoting MDSC apoptosis (1 μg/mL once a day for 14 days, i.p.)	Sun et al. (2019)
			Ovarian cancer	*In vitro* and *in vivo*	Inhibiting the polarization of M2 macrophages and promoting M1 polarization by inhibiting the PI3K/AKT/NF-κB pathways (0.15 mg/kg of 0.2 mL once a day for 14 days, i.p.)	Hu (2020)
			Tumor-bearing mice	*In vivo*	Downregulation of Tregs, IL-10, TGF-β, and VEGF levels (0.15 mg/kg once a day for 7 days, i.p.)	Liu et al. (2013)
	Oridonin	*Rabdosia rubescens*	Breast cancer	*In vitro* and *in vivo*	Inhibiting Treg differentiation by decreasing TGF-β receptor expression (5 mg/kg once every 3 days for 24 days, i.p.)	Guo et al. (2020)
	Sclareol	*Salvia sclarea*	Breast cancer	*In vivo*	Decreasing the rate of tumor growth by increasing IFN-γ and decreasing IL-4 (7.85 µg once a day for 6 days)	Shokoofe et al. (2010)
	Paclitaxel	*Taxus brevifolia* Nutt	Advanced non-small-cell lung cancer	Clinical	Inducing Treg apoptosis by upregulating CD95 (30 ng/mL)	Zhang et al. (2008)
			Melanoma	*In vitro*	Suppressing tumor by inhibiting MDSC differentiation into dendritic cells (0.2 and 1 nM)	Tillmann et al. (2012)
	Carnosol	*Rosmarinus officinalis* L.	Fibrosarcoma	*In vivo*	Reducing the relative level of immunosuppressive Tregs and shifting toward increasing IFN-γ expression (5 and 10 mg/kg, respectively, once a day, i.p.)	Maryam et al. (2015)
Sesquiterpenoids	Artemisinin	*Artemisia annua* L.	Breast cancer	*In vitro* and *in vivo*	Inhibiting the *in vivo* growth of breast cancer by promoting T-cell activation and inhibiting the expression of Tregs and MDSCs in the tumor microenvironment (100 mg/kg once a day for 20 days, i.p.)	Cao et al. (2019)
	Fraxinellone	*Dictamnus dasycarpus* Turcz	Oral squamous cell carcinoma	*In vivo*	Suppressing tumor by promoting M1 macrophage polarization (50 and 100 mg/kg, respectively)	Zou et al. (2023)
	Tehranolide	*Artemisia annua* L.	Breast cancer	*In vivo*	Inhibiting tumor growth by reducing the number of CD4^+^ CD25^+^ FoxP3^+^ T lymphocytes (5.64 µg once a day for 6 days, i.p.)	Noori et al. (2010)
					Suppressing tumors by attenuating CD4^+^ CD25^+^ FoxP3^+^ Treg-mediated immune suppression and eliciting persistent antitumor immunity against cancer (5.64 μg once a day for 25 days, i.p.)	Noori et al. (2009)

### 3.6 Quinones and other compounds

**FIGURE 7:**
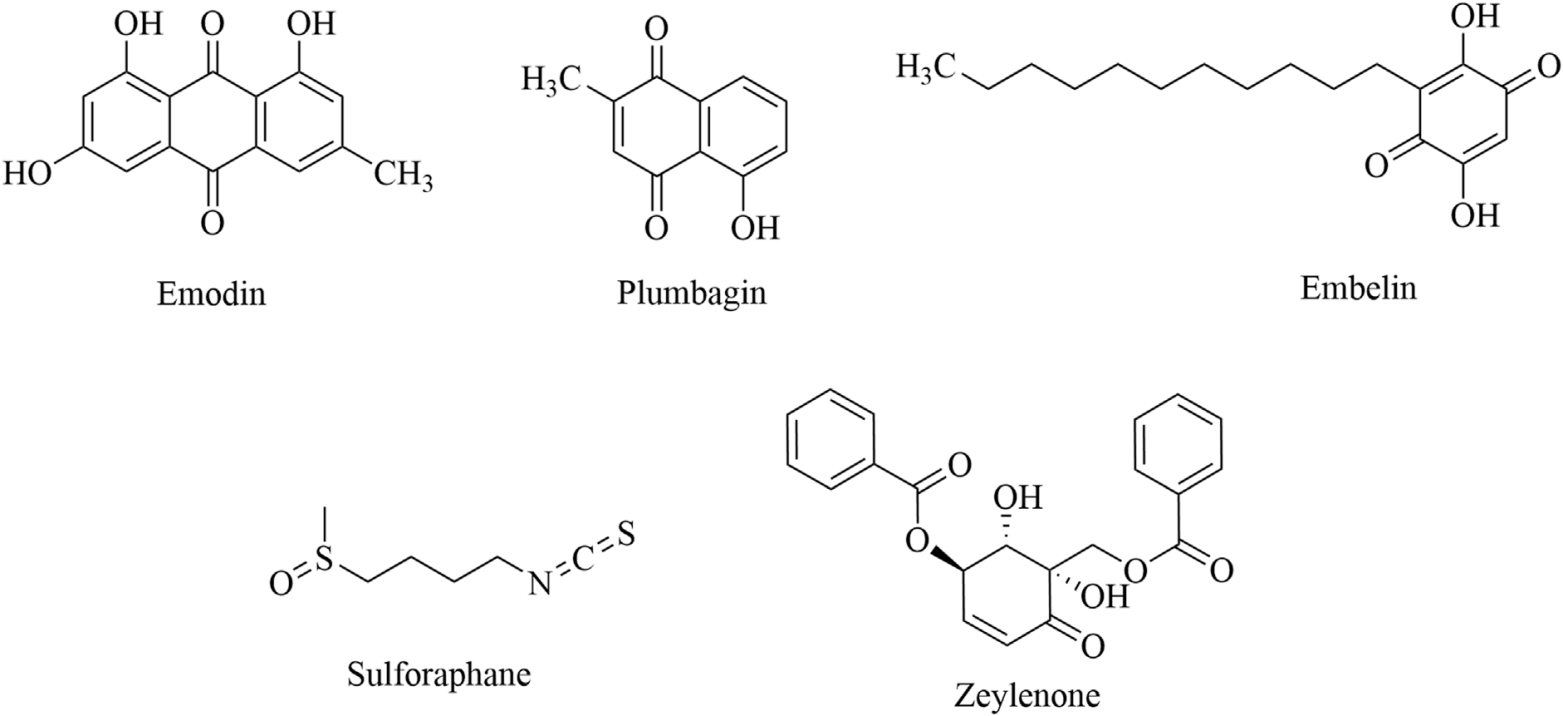
Structural formulas of natural quinones and other compound products.

Quinones that target the regulation of immune cells can be classified into three categories: anthraquinone, naphthoquinone, and benzoquinone. Emodin inhibits Treg migration by inhibiting the expression of CCR4, VEGF-C, and MMP-9 in tumor tissue (Ruan, et al., 2014; Ruan et al., 2013; Wang, 2018). Plumbagin can reduce the inhibition of T cells by inhibiting the differentiation of MDSCs and the expression of CCL2 and IL-6, ultimately exerting an antitumor effect (Chen, 2018). Sulforaphane (SFN) induces MDSC apoptosis by promoting the expression of IL12RB2 and enhancing the infiltration of CD8^+^ T cells (Chen, 2022). Zeylenone promotes the polarization of M1 macrophages and inhibits M2 polarization by inhibiting the PI3K/AKT signaling pathway, thereby reducing the activity of colorectal cancer cells and promoting their apoptosis (Li D. et al., 2024). Detailed information is presented in Table 6, and the structural formula is shown in Figure 7.

**TABLE 6:** Natural antitumor quinones and other compounds, their sources, experimental models, and mechanisms of targeting regulatory immune cells.

Category	Name	Source	Tumor type	Clinical/experimental	Targeted regulatory immune cell mechanisms	Reference
Anthraquinone	Emodin	*Polygonum cuspidatum* Sieb. et Zucc. and *Rheum palmatum* L.	Colon cancer	*In vivo*	Suppressing tumors by inhibiting the immunosuppressive function of Tregs (100 mg/kg once a day for 14 days, i.g.)	Ruan et al. (2014)
			Colon cancer	*In vivo*	Inhibiting Treg migration by decreasing CCR4 expression (100 mg/kg once a day for 14 days, i.g.)	Ruan et al. (2013)
			Colon cancer	*In vivo*	Inhibiting Treg migration by inhibiting the expression of VEGF-C and MMP-9 in tumor tissue (100 mg/kg once a day for 14 days, i.p.)	Wang (2018)
Naphthoquinone	Plumbagin	*Plumbago indica* L.	Pancreatic cancer	*In vivo*	Reducing the inhibition of T cells by inhibiting the differentiation of MDSCs and the expression of CCL2 and IL-6 (2 mg/kg once a day for 14 days, i.p.)	Chen (2018)
Benzoquinone	Embelin	*Embelia ribes* Burm. f	Pancreatic cancer	*In vitro* and *in vivo*	Inhibiting MDSC and Treg invasion by regulating the p53 pathway and STAT3 pathway (50 mg/kg once a day for 14 days, i.p.)	Peng (2014)
Isothiocyanates	Sulforaphane	Broccoli and cabbage	Colorectal cancer	*In vitro* and *in vivo*	Inducing MDSC apoptosis by promoting the expression of IL12RB2 (50 mg/kg once a day for 12 days, i.p.)	Chen (2022)
Cyclohexene oxide	Zeylenone	*Uvaria grandiflora* Roxb	Colorectal cancer	*In vitro* and *in vivo*	Promoting the polarization of M1 macrophages by inhibiting the PI3K/AKT signaling pathway (30 mg/kg every other day for 14 days, i.p.)	Li X. et al. (2024)

## 4 Conclusion and prospects

**FIGURE 8:**
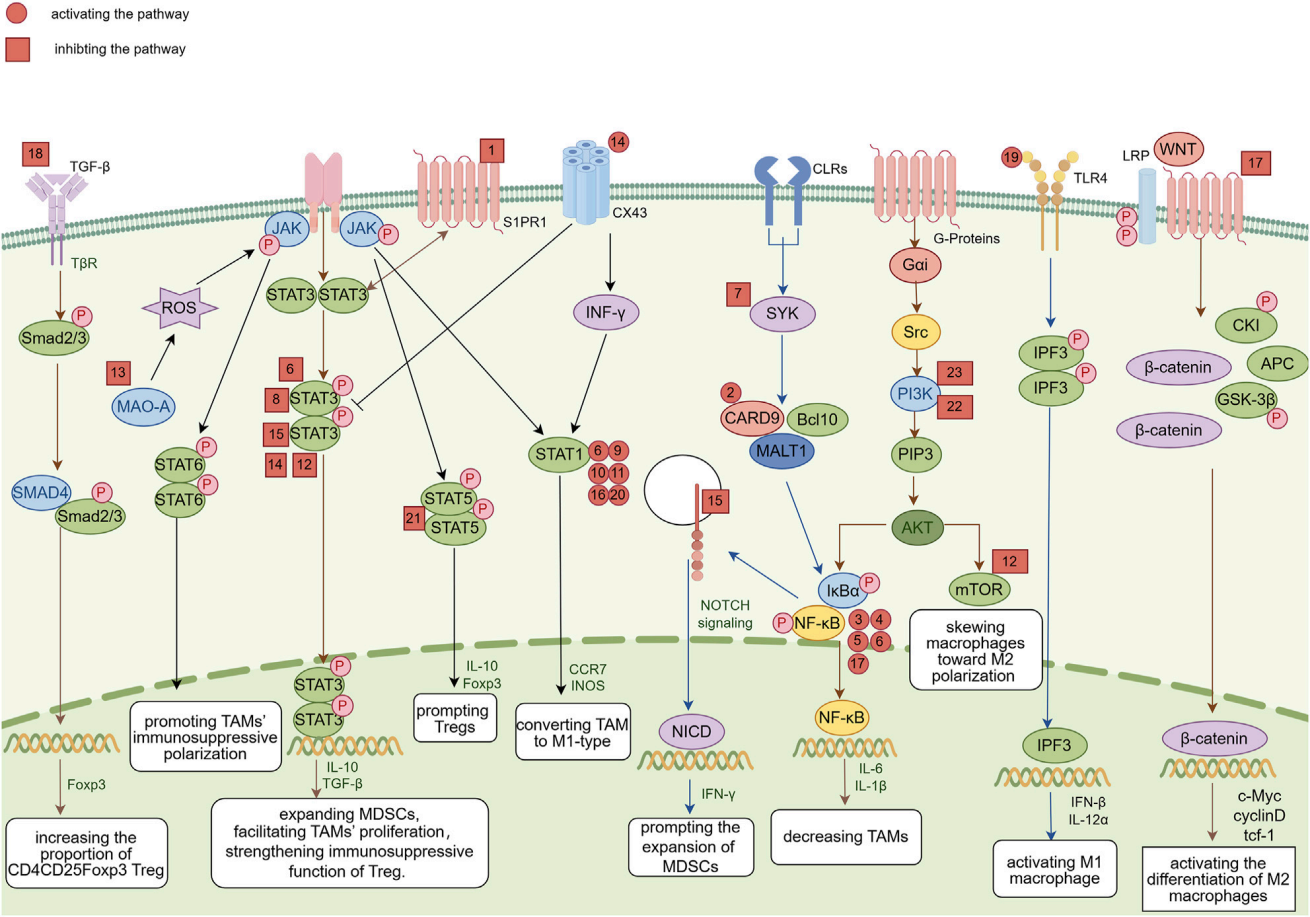
Overview of the molecular mechanisms of representative natural products, which are indicated by corresponding numbers. 1: Astragalus. 2: *Ganoderma lucidum* polysaccharide. 3: *Armillaria mellea* polysaccharides. 4: Polysaccharides from *Cistanche deserticola*. 5: *Lachnum* polysaccharide. 6: Polysaccharide from *Ilex asprella*. 7: Resveratrol. 8: Epigallocatechin-3-gallate. 9: Wogonin. 10: Baicalein. 11: Apigenin. 12: Cardamonin. 13: Curcumin. 14: Dioscin. 15: Ginsenoside Rg3. 16: Vitexin. 17: Sanguinarine. 18: Solanine. 19: Sophoridine. 20: 23-Hydroxybetulinic acid. 21: Ursolic acid. 22: Triptolide. 23: Zeylenone. Image was created using Figdraw.com.

Regulatory immune cells strongly influence the tumor microenvironment. Tregs, Bregs, MDSCs, DCregs, and TAMs regulate immune responses through various mechanisms, affecting the occurrence, development, and therapeutic effects of tumors. In this article, we reviewed the classification, function, and mechanism of action of these regulatory immune cells in the tumor microenvironment and investigated the potential of natural products in regulating these immune cells. Natural products exhibit significant immunomodulatory effects, influencing the function of regulatory immune cells and promoting antitumor immune responses (Figure 8).

In addition, the antineoplastic agent paclitaxel has gained importance as a promising agent for reducing the number of Tregs in the peripheral blood of non-small cell lung cancer patients at a dose of 30 ng/mL (Zhang et al., 2008). Furthermore, additional treatment with 4 mg of lentinan once daily, administered through intramuscular injection for 12 weeks to non-small-cell lung cancer patients who received NP (combination of vinorelbine and cisplatin) first-line chemotherapy, can prolong the survival benefits of progression-free survival (PFS) or even OS by downregulating the percentage of Tregs in the peripheral blood and leading to a shift in the inflammatory status from Th2 to Th1 (Wang et al., 2018). By treating with different doses of APS (10, 50, 100, 150, and 200 μg/mL) for 24, 48, and 72 h, the number of Tregs decreased in a dose-dependent manner in the peripheral blood of hepatocellular carcinoma (HCC) patients. It indicated that the application of APS in the tumor microenvironment could enhance the antitumor effects of immunotherapy approaches, thereby potentially increasing the survival rate in patients with HCC (Li, et al., 2012).

Despite the substantial amount of literature exploring natural products, most studies continue to focus on the mechanisms of drug action, with relatively few studies addressing the bioavailability and toxicity of these natural compounds. Furthermore, research on the clinical development of natural products is also limited. In the future, more systematic clinical trials should be conducted to verify the efficacy and safety of natural products and investigate the combination of natural products and existing tumor treatment methods, such as immune checkpoint inhibitors, chemotherapy, and radiotherapy, which may synergistically affect and improve treatment effectiveness. Researchers should also identify and develop more natural products with immunomodulatory functions and evaluate their ability to treat tumors, thereby promoting their application in cancer treatment.

To summarize, research on regulatory immune cells and natural products can improve the efficacy of cancer treatment. In-depth research on the mechanism of action, clinical applications, combination therapy, and the development of new natural products can further enhance the effectiveness of tumor treatment and improve patient prognosis.
